# Theabrownin ameliorates liver inflammation, oxidative stress, and fibrosis in MCD diet-fed C57BL/6J mice

**DOI:** 10.3389/fendo.2023.1118925

**Published:** 2023-01-18

**Authors:** Qingcai Zhen, Qijian Liang, Hongchun Wang, Yan Zheng, Zhongting Lu, Chunyong Bian, Xiulan Zhao, Xin Guo

**Affiliations:** ^1^ Department of Nutrition and Food Hygiene, School of Public Health, Cheeloo College of Medicine, Shandong University, Jinan, Shandong, China; ^2^ Shandong Engineering Research Center of Biomarker and Artificial Intelligence Application, Jinan, Shandong, China; ^3^ Department of Clinical Laboratory, Qilu Hospital of Shandong University, Jinan, Shandong, China; ^4^ Research Center of Translational Medicine, Jinan Central Hospital Affiliated to Shandong First Medical University, Jinan, China; ^5^ Institute of Toxicology, School of Public Health, Cheeloo College of Medicine, Shandong University, Jinan, Shandong, China

**Keywords:** theabrownin, NASH, inflammation, fibrosis, ROS

## Abstract

**Introduction:**

Nonalcoholic steatohepatitis (NASH), also known as metabolic steatohepatitis, is a clinical syndrome with pathological changes like alcoholic hepatitis but without a history of excessive alcohol consumption. NASH is closely related to metabolic disorders such as obesity, insulin resistance, type 2 diabetes mellitus, and hyperlipidemia. Its main characteristics are hepatocyte steatosis with hepatocyte injury and inflammation. In severe cases, it can develop into liver cirrhosis. At present, there is no special treatment for NASH. Theabrownin (TB) is the main pigment substance in fermented tea. Theabrownin has beneficial effects on lipid metabolism and intestinal flora. However, the effect of theabrownin on NASH has not been studied.

**Methods:**

This study was aimed at exploring the effects of theabrownin from Fuzhuan brick tea on NASH. 8-week-old mice were randomly assigned to three groups and fed with chow diet (CD), methionine and choline sufficient (MCS) diet (MCS Ctrl), which is a Methionine/choline deficient (MCD) control diet, and MCD diet. After 5 weeks of feeding, the MCD group mice were randomly divided into two groups and were gavaged with double distilled water (MCD Ctrl) or theabrownin (MCD TB) (200mg/kg body weight, dissolved in double distilled water) every day for another 4 weeks respectively, while continuing MCD diet feeding.

**Results:**

We found that theabrownin treatment could not improve liver mass loss and steatosis. However, theabrownin ameliorated liver injury and decreased liver inflammatory response. Theabrownin also alleviated liver oxidative stress and fibrosis. Furthermore, our results showed that theabrownin increased hepatic level of fibroblast growth factor 21 (FGF21) and reduced the phosphorylation of mitogen-activated protein kinase p38 in MCD diet-fed mice.

## 1 Introduction

With the globalization of obesity and its related metabolic syndrome, nonalcoholic fatty liver disease (NAFLD) has become an important cause of chronic liver disease in developed countries such as Europe and the United States and rich regions in China (1). According to the degree of pathological changes and whether the pathological liver tissue is accompanied by inflammatory reaction and fibrosis, NAFLD can be divided into simple fatty liver, non-alcoholic steatohepatitis (NASH) and NASH-related cirrhosis (2). The pathogenesis of NAFLD is complex, which have not been fully elucidated by the current research. A well-known " multiple hit" hypothesis is proposed for explaining the onset and development of NAFLD (3). According to this theory, NAFLD is caused by many factors, including hormones secreted by adipose tissue, insulin resistance, dietary factors, intestinal flora, and genetic and epigenetic factors. Physical inactivated lifestyle and over-eating related unhealthy eating habits will lead to liver fat accumulation. Excess fat storage in the peripheral and liver leads to inflammation of adipose tissue and liver. The liver, peripheral adipose tissue and intestine interact through cytokines, while the liver is at the central stage of metabolic regulation. Lipids, particularly free fatty acids derived from the periphery, overflow in hepatocytes, leading to mitochondrial and peroxisomal dysfunction and enhanced oxidative stress. The enhanced reactive oxygen species (ROS) causes hepatocyte damage, which triggers macrophage infiltration in liver. When this inflammatory process becomes chronic, further metabolic deterioration and fibrosis will follow (4–6). At present, the prevalence of NASH in the population is 3-5% (1, 3, 4). So far, there are currently no approved pharmacological therapies for NASH (7). Therefore, it is urgent to explore new targets and methods to prevent NASH and liver fibrosis. Looking for food functional factors to reduce liver inflammation and fibrosis may be a potential effective method to prevent and treat NASH.

Fibroblast growth factor 21 (FGF21), a metabolic regulator, is a peptide hormone (8), which can be produced by liver, fat tissue and pancreas (9). Recent studies have found that FGF21 is related to the pathogenesis and development of NAFLD. FGF21 can inhibit the progression of nonalcoholic fatty liver disease (10). A study has shown that exercise can stimulate the production of FGF21 in muscle and subsequently promote the lipophagy in the liver, thus playing an important role in improving NAFLD (11). Astaxanthin, a nutrient-related substance, can improve liver mitochondrial function and ameliorate NAFLD through up-regulating FGF21/PGC1 α pathway (12). Supplementation of *Bifidobacterium* can improve hepatic steatosis and steatohepatitis *via* elevating expressions of the receptors of FGF21 to increase the sensitivity of FGF21 (13). NAFLD can also be improved by subcutaneous injection of FGF21 (14).

Theabrownins (TB), the main pigment substance in fermented tea (15), are water-soluble phenolic compounds (15, 16). It is generally considered to be formed by further oxidative polymerization of tea polyphenols Theaflavins (TFs), Thearubigins (TRs), and its color is brown or maroon (17, 18). Fuzhuan brick theabrownin is a kind of theabrownin, which extracted from Fuzhuan brick tea. A previous study showed that Fuzhuan tea can significantly alleviate liver lipid deposition and inflammation, as well as improving intestinal flora in rats fed with high-fat diet (19). Fuzhuan tea supplementation can also improve arterial stiffness in mice (20). Fuzhuan brick tea also contains probiotics, such as *Eurotium cristatum*, which can improve intestinal flora in obesity and metabolic disorder (21, 22). Theabrownin extracted from Fuzhuan tea can also ameliorate disorders of lipid and glucose metabolism in obese mice (23).

Although there is many evidence showed that theabrownin is associated with improving obesity-related diseases, the effect of theabrownin on NAFLD has not been fully understood. More importantly, the effect of theabrownin extracted from Fuzhuan brick tea on NASH and its mechanism has not been studied. Therefore, in this study, we investigated the effect of theabrownin extracted from Fuzhuan brick tea on MCD diet-induced NASH mice and the mechanism under it.

## 2 Materials and methods

### 2.1 Animal experiments

4-week-old C57BL/6J male mice were obtained from Beijing Vital River Laboratory Animal Technology Corporation (Beijing, China) and were housed at 23°C, with 50% humidity and on a 12 h light−dark cycle. After more than a month of adaptive feeding, 8-week-old mice were randomly assigned to three groups and fed with chow diet (CD), methionine and choline sufficient (MCS) diet (MCS Ctrl), which is a MCD control diet, and MCD diet. MCS and MCD diet are products of Research Diets, Inc (New Brunswick, NJ). In this experiment, we fed mice with MCD diet for 5 weeks to induce NASH model. After 5 weeks of feeding, the MCD group mice were randomly divided into two groups and were gavaged with double distilled water (MCD Ctrl) or theabrownin (MCD TB) (Lander Biotech, Xi’an, China) (200mg/kg body weight, dissolved in double distilled water) every day for another 4 weeks respectively, while continuing MCD diet feeding. CD group contains 5 mice, MCS Ctrl group contains 11 mice, MCD Ctrl group and MCD TB group contain 7 mice each. Body weights were recorded once a week during the experimental period. At the end of experiments, mice were sacrificed under anesthesia and blood samples and the liver were collected. Bloods samples were centrifuged at 12000 rpm and 4°C for 5 min. One part of liver tissue was fixed in the 10% neutral formalin for pathological observation and the rest of liver tissue was frozen in the liquid nitrogen and reserved at -80℃ for further research. All procedures were approved by the Institutional Animal Care and Use Committee at Shandong University and performed in conformance with the guide.

### 2.2 Measurement of TG

The levels of Triglyceride (TG) in serum and liver tissues were determined by Liquid Sample Triglyceride (TG) Content Assay Kit (Applygen Technologies Inc, #E1003) and High Fatty Sample Triglyceride (TG) Content Assay Kit separately (Applygen Technologies Inc, #E1025). The TG level of serum was determined following the manufacturer's instructions. Liver tissues were homogenized in lysis solution, and then let the lysate stand for 10 minutes. One part of the lysate was heated at 70°C for 10 minutes and then centrifuged at 2000rpm at room temperature for 5 minutes. The supernatant was used for enzymatic determination following the manufacturer's instructions. The remaining part of the lysate can be quantified by the BCA protein quantitative kit (Beyotime Biotechnology, #P0010). The final levels of liver TG were normalized by protein concentration.

### 2.3 Measurement of ALT and AST

The serum levels of Alanine aminotransferase (ALT) and Alanine aminotransferase (AST) were determined by using ALT kit (Nanjing Jiancheng Biotechnology, #C009-2-1) and AST kit (Nanjing Jiancheng Biotechnology, #C010-2-1) respectively.

### 2.4 Measurement of MDA

Liver tissues were homogenized in phosphate-buffered saline (PBS) then centrifuged at 12000 rcf and 4 °C for 15 minutes. The supernatant was used for determination of Malondialdehyde (MDA) following the manufacturer's instructions. The remaining part of the lysate can be quantified by BCA protein quantitative kit (Beyotime Bio-technology, #P0010). The final levels of liver MDA were normalized by protein concentration.

### 2.5 Detection of liver reactive oxygen species level

Liver tissues were fixed in 10% neutral formalin, dehydrated and paraffin-embedded and cut into 5 μm thick sections. The sections of liver tissue were incubated with dihydroethidium solution (DHE, 1 μmol/L, Beyotime Biotechnology, # S0063) at 37°C in the dark for 30 min following the manufacturer's instructions. The fluorescence was measured under the excitation of 580 nm using a fluorescence microscope (EVOS FL, Thermo Fisher Scientific) (24).

### 2.6 Histological analysis

Liver tissues were fixed in 10% neutral formalin, dehydrated and paraffin-embedded and cut into 5 μm thick sections for hematoxylin and eosin (H&E), Immunohistochemistry (IHC), Masson’s Trichrome and Picrosirius Red staining. Hepatic fibrosis was analyzed by Masson’s Trichrome, Picrosirius Red staining, and IHC staining for smooth muscle actin (SMA). After that, staining was observed under microscope. For IHC, the antibodies contained F4/80 (1:400) (Cell Signaling Technology, #70076), Ly-6G (1:400) (Santa Cruz Biotechnology, #sc-53515), IL-1β (1:400) (Cell Signaling Technology, #12242), SMA (1:400) (Santa Cruz Biotechnology, #sc-53142), Fibroblast growth factor 21 (Affinity Biosciences, #DF8947), Phospho-p44/42 MAPK (Erk1/2) (Thr202/Tyr204) (Cell Signaling Technology, #4370), p44/42 MAPK (Erk1/2) (137F5) (Cell Signaling Technology, #4695). Image J software (National Institutes of Health, Bethesda, Maryland) was used for quantification.

### 2.7 Western blot analysis

Lysates prepared from frozen liver samples were used for western blot. The level of phospho-NF-κB p65 (Ser536) (Cell Signaling Technology, #3033), NFκB p65 (Cell Signaling Technology, #8214), the stress-activated protein kinase/Jun-amino-terminal kinase (SAPK/JNK) (Cell Signaling Technology, #9252), phospho-SAPK/JNK(Thr183/Thr185) (Cell Signaling Technology, #9251), IL-1β (Cell Signaling Technology, #12242), Phospho-p38 MAPK (Thr180/Tyr182) (Cell Signaling Technology, #4511), p38 MAPK (Cell Signaling Technology, #8690), FGF21 (Affinity Biosciences, #DF8947), Collagen 1 a 1 (Santa Cruz Biotechnology, # sc-293182),Collagen 3 a 1 (Santa Cruz Biotechnology, #sc-271249), Smooth muscle ac-tin (Santa Cruz Biotechnology, #sc-53142) and Tubulin (Cell Signaling Technology, #2125) were analyzed as described (25, 26).

### 2.8 Real-time quantitative polymerase chain reaction

Total RNA was isolated from liver tissues by using a commercial kit (RNAeasy™ Animal RNA Isolation Kit with Spin Column, Beyotime Biotechnology, # R0027), and 1 μg total RNA was reversed transcribed to cDNA with First-Strand cDNA Synthesis Kit (Accurate Biology, #AG11728) according to instruction book suggested by the manufacturer. Real-time PCR was performed with LightCycler 480 Real-Time PCR System (Accurate Biology, #AG11701) following the manufacturer's instructions. The sequences of primers used in this study are listed in **Table 1**.

**Table 1:** Primers for RT-PCR.

Genes	Primers	Sequences
ACC1	Forward	CGCTCGTCAGGTTCTTATTG
	Reverse	TTTCTGCAGGTTCTCAATGC
FAS	Forward	GGAGGTGGTGATAGCCGGTAT
	Reverse	TGGGTAATCCATAGAGCCCAG
SREBP1c	Forward	GGAGCCATGGATTGCACATT
	Reverse	GGCCCGGGAAGTCACTGT
CPT1A	Forward	CTACATCACCCCAACCCATATT
	Reverse	GATCCCAGAAGACGAATAGGTT
MCP-1	Forward	GAAAACTGAGGCACCAAGGG
	Reverse	AGGTGGAGAGTGATGTTGGG
ICAM-1	Forward	GCTACCATCACCGTGTATTCG
	Reverse	AGGTCCTTGCCTACTTGCTG
COL1A1	Forward	CGGATAGCAGATTGAGAACATCCG
	Reverse	CGGCTGAGTAGGGAACACACA
FN	Forward	CGGAGAGAGTGCCCCTACTA
	Reverse	CGATATTGGTGAATCGCAGA
TGFβ1	Forward	GACTCTCCACCTGCAAGACCAT
	Reverse	GGGACTGGCGAGCCTTAGTT
FGF21	Forward	ATGGAATGGATGAGATCTAGAGTTGG
	Reverse	TCTTGGTGGTCATCTGTGTAGAGG
36B4	Forward	GGCTGACTTGGTTGCTTTGG
	Reverse	AGCAAAGGAAGAGTCGGAGG

### 2.9. Statistical analysis

All experimental numeric data are presented as mean ± standard error of the mean (SEM). One-way ANOVA and least significance difference (LSD) method as a suitable *post-hoc* test was used to determine the differences among groups by using SPSS 26 (IBM, SPSS, USA) and a p-value of < 0.05 was considered statistically significant.

## 3 Results

### 3.1 Theabrownin does not change body weight, liver weight and liver lipid deposition in MCD diet-fed mice

**Figure 1:**
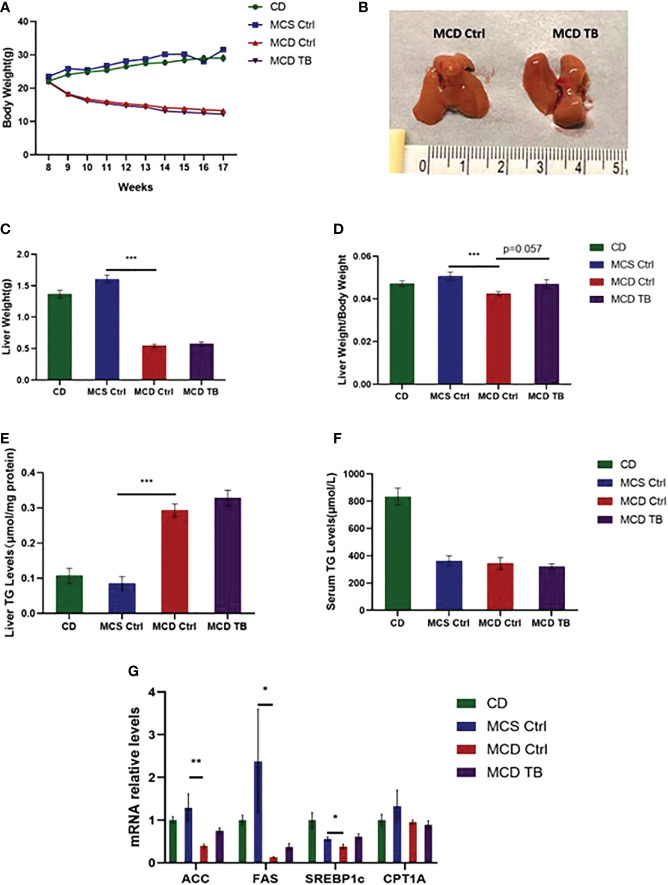
Theabrownin does not change body weight, liver weight and liver lipid deposition in MCD diet-fed mice. **(A)** The change of body weight. **(B)** Liver morphology. **(C)** Liver weight. **(D)** Liver coefficient. **(E)** Liver TG level. **(F)** Serum TG level. **(G)** mRNA levels of ACC, FAS, SREBP1c, and CPT1A. n=5-11 mice per group. The data are mean ± s.e. (error bars). *p < 0.05, **p < 0.01, ***p < 0.001, MCS Ctrl vs MCD Ctrl.

Liver steatosis is the initial manifestation of NAFLD. To investigate the effect of theabrownin on fat deposition in NASH mice, 8 weeks old mice were fed with CD, MCS or MCD diet for 5 weeks. In MCD diet feeding, mice were randomly divided into two groups and were gavaged with water or theabrownin for another 4 weeks separately. Body weight in MCD diet-fed mice significantly decreased compared to mice fed with MCS diet and CD (**Figure 1A**). Theabrownin didn’t influence the body weight of MCD-fed mice compared to control group (**Figure 1A**). In addition, theabrownin could not change the liver size (**Figure 1B**), liver weight (**Figure 1C**) and liver coefficient (**Figure 1D**), compared to MCD diet-fed control mice. Mice fed with MCD diet develop severe hepatic steatosis, which is manifested by increased hepatic triglyceride levels (27, 28). Similarly, in the current study, MCD diet can significantly increase the content of triglyceride (TG) in liver of mice (**Figure 1E**). However, theabrownin cannot reduce the level of TG in liver (**Figure 1E**), nor can it reduce the content of triglyceride in serum (**Figure 1F**). Moreover, the mRNA levels of lipogenic genes such as acetyl-CoA carboxylase (ACC), fatty acid synthase (FAS), and sterol regulatory element-binding protein-1c (SREBP1c) in the liver of mice fed with MCD diet were lower than those fed with MCS diet (**Figure 1G**). It indicated that rather than the increase of *de novo* lipogenesis in liver, fats from adipose tissue lipolysis transported to liver may contribute to the high TG levels in MCD diet-fed mice (29). Consistently, in our study, almost no white adipose tissue was found in mice fed with MCD diet. It suggested that adipose tissue lipolysis may happen in MCD diet-fed mice. In addition, theabrownin did not change the mRNA levels of lipogenic genes in MCD diet-fed mice (**Figure 1G**). The mRNA level of carnitine palmitoyltransferase 1A (CPT1A), which was a key enzyme in for fatty acid oxidation, was not shown difference between MCS and MCD diet-fed mice and was not altered by administration of theabrownin (**Figure 1G**). This data indicated that theabrownin did not alleviate loss of liver mass and liver steatosis in NASH mice.

### 3.2 Theabrownin improves liver injury and inflammation in MCD diet-fed mice

**Figure 2:**
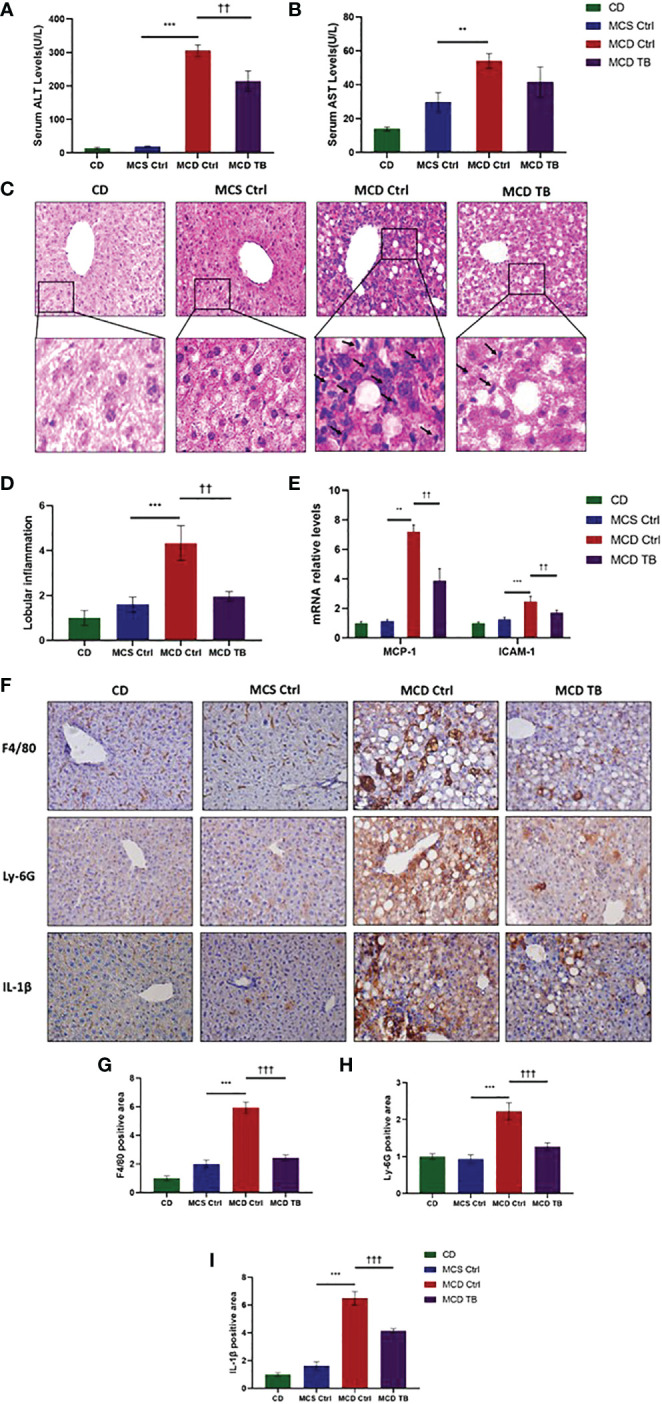
Theabrownin improves liver injury and inflammation in MCD diet-fed mice. **(A)** Serum ALT level. **(B)** Serum AST level. **(C)** Liver section for H&E staining. **(D)** Quantification of lobular inflammation. **(E)** mRNA levels for MCP-1 and ICAM-1. **(F)** Liver section for IHC with F4/80, Ly-6G, and IL-1β. **(G–I)** Quantification of positive area for F4/80, Ly-6G, and IL-1β. n=5-11 mice per group. The data are mean ± s.e. (error bars). **p < 0.01, ***p < 0.001, MCS Ctrl vs. MCD Ctrl; ††, p<0.01, †††, p<0.001 MCD Ctrl vs. MCD TB.

**Figure 3:**
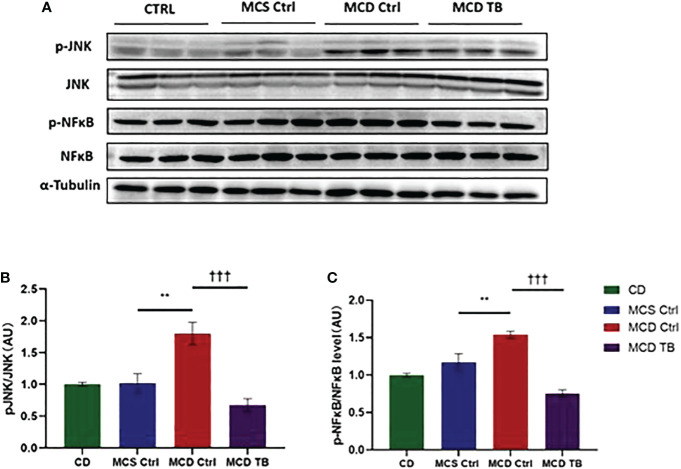
Theabrownin reduces liver pro-inflammatory response. **(A)** The phosphorylation and protein levels of NFκB p65 and JNK. **(B)** Quantification of JNK phosphorylation. **(C)** Quantification of NFκB p65 phosphorylation. AU, arbitrary units. n=5-11 mice per group. The data are mean ± s.e. (error bars). **p < 0.01, MCS Ctrl vs. MCD Ctrl; †††, p<0.001 MCD Ctrl vs. MCD TB.

Although theabrownin has no effect on hepatic fat accumulation in MCD diet-fed mice, we wanted to figure out whether theabrownin influenced liver injury and inflammation in NASH. MCD diet can induce severe liver injury and inflammatory response (30). To determine whether theabrownin influenced liver function, we measured AST and ALT in serum. Consistent with previous studies, the ALT and AST levels in serum were significantly increased in MCD diet-induced NASH mice, while theabrownin reduced the high serum level of ALT (**Figure 2A**). Although theabrownin has no obvious effect on serum AST level, the decreasing trend of AST level was seen in MCD diet-fed mice treated with theabrownin (**Figure 2B**). The results indicated that theabrownin could alleviate liver injury. Except fat accumulation, NASH is also characterized by liver inflammation (6). Next, we investigated the effect of theabrownin on NASH related inflammation. Consistent with the above result, H&E staining of liver sections shows that theabrownin did not alleviate liver steatosis in MCD diet-fed mice. However, theabrownin significantly reduced the lobular inflammation in liver (**Figures 2C, D**). Moreover, IHC staining of the macrophage marker F4/80 and neutrophils marker Ly-6G showed that theabrownin significantly decreased macrophage and neutrophils infiltration (**Figures 2F–H**). Real-time Quantitative polymerase chain reaction assay showed that theabrownin significantly reduced the mRNA levels of pro-inflammatory genes monocyte-chemoattractant protein 1 (MCP1) and intercellular adhesion molecule 1 (ICAM-1) (**Figure 2E**). It further proved that theabrownin reversed the immune cells infiltration induced by MCD diet. We further confirmed the activation of NFκB and JNK by western blot analysis. Theabrownin reduced the increase of phosphorylation of NFκB p65 and JNK induced by MCD diet in liver (**Figures 3A–C**). Interleukin 1 beta (IL-1β), which is a key mediator of the inflammatory response, sends signals through IL-1 receptors widely expressed in different liver cell subsets to promote liver inflammation and fibrosis (6). In MCD diet-induced NASH mice, the level of IL-1β was remarkably enhanced, while theabrownin significantly reduced it (**Figures 2F , I**). It indicated that theabrownin decreased inflammatory response in NASH mice. These results suggest that theabrownin can improve liver injury and hepatic inflammation in MCD diet-fed mice.

### 3.3 Theabrownin reduces hepatic oxidative stress and fibrosis in MCD diet-fed mice

**Figure 4:**
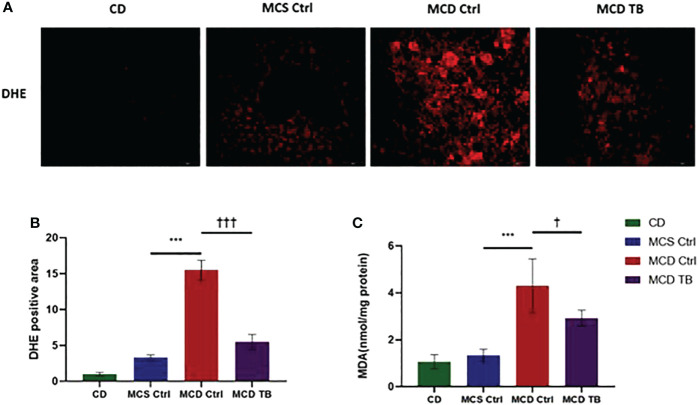
Theabrownin reduces hepatic oxidative stress in MCD diet-fed mice. **(A)** Liver section for ROS generation (DHE level). **(B)** Quantification of positive area for DHE. **(C)** Liver MDA level. n=5-11 mice per group. The data are mean ± s.e. (error bars). ***p < 0.001, MCS Ctrl vs. MCD Ctrl. †, p<0.05, †††, p<0.001 MCD Ctrl vs. MCD TB.

**Figure 5:**
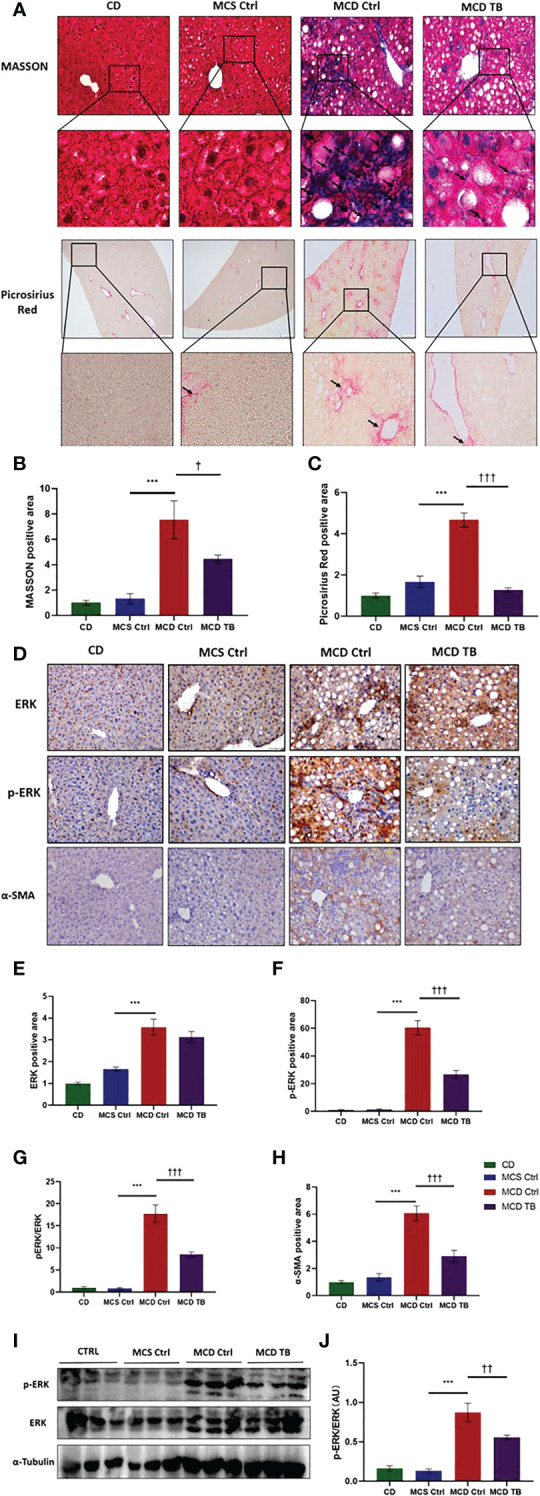
Theabrownin reduces hepatic fibrosis in MCD diet-fed mice. **(A)** Liver section for Masson and Picrosirius Red staining. **(B, C)** Quantification of positive area for Masson and Picrosirius Red staining. **(D)** IHC for ERK, ERK phosphorylation, and α-SMA. **(E)** Quantification of IHC positive area for ERK. **(F)** Quantification of IHC positive area for ERK phosphorylation. **(G)** Quantification of ERK phosphorylation from IHC (normalized by total ERK). **(H)** Quantification of positive area for α-SMA. **(I)** Protein levels of ERK and ERK phosphorylation. **(J)** Quantification of ERK phosphorylation from western blot. AU, arbitrary units. n=5-11 mice per group. The data are mean ± s.e. (error bars). ***p < 0.001, MCS Ctrl vs. MCD Ctrl. †, p<0.05, ††, p<0.01, †††, p<0.001 MCD Ctrl vs. MCD TB.

**Figure 6:**
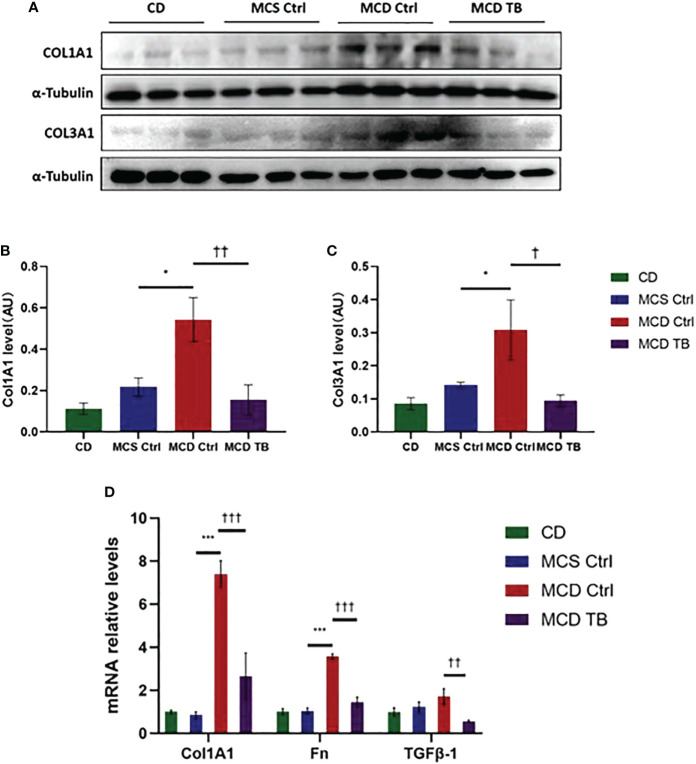
Theabrownin reduces hepatic levels of collagen and glycoprotein in MCD diet-fed mice. **(A)** Protein levels of COL1A1 and COL3A1. **(B)** Quantification of COL1A1 level. **(C)** Quantification of COL3A1 level. **(D)** mRNA levels of COL1A1, Fn, and TGFβ-1. AU, arbitrary units. n=5-11 mice per group. The data are mean ± s.e. (error bars). *p < 0.05, ***p < 0.001, MCS Ctrl vs. MCD Ctrl. †, p<0.05, ††, p<0.01, †††, p<0.001 MCD Ctrl vs. MCD TB.

Oxidative stress plays a crucial role in the pathogenesis and progression of NASH (31). Next, we investigated the effect of theabrownin on oxidative stress in the liver of NASH mice. Dihydroethidium (DHE) is a fluorescent probe for the detection of ROS generation. ROS production in the liver of MCD diet-fed mice much higher than that of MCS diet fed mice (**Figures 4A, B**). Theabrownin significantly reduced the ROS production in MCD diet-fed mice (**Figures 4A, B**). The levels of MDA in liver, which is considered a biomarker for oxidative damage of lipids, was dramatically increased in MCD diet fed mice, compared to MCS diet-fed mice, while theabrownin significantly decreased it in MCD diet-fed mice (**Figure 4C**). The results indicated that the theabrownin could downregulate oxidative stress in the NASH mice. Fibrosis is a hallmark of NASH (32). Next, we investigated the effect of theabrownin on NASH related fibrosis. Masson staining and Picrosirius Red staining in liver sections showed that MCD diet induced to develop liver fibrosis in mice, which can be alleviated by theabrownin (**Figures 5A–C**). The activation of extracellular signal-regulated kinase (ERK), which is a key component of MAPK signaling pathway, is associated with the development of liver fibrosis (33). MCD diet significantly increased the expression and the phosphorylation of ERK, while theabrownin significantly reduced the phosphorylation of ERK in liver, as shown from IHC staining (**Figures 5D–G**) and western blot (**Figures 5I, J**). Theabrownin also obviously reduced the high α-SMA level induced by MCD diet (**Figures 5D, H**). In addition, theabrownin significantly reduced the high mRNA levels of fibrogenesis genes collagen type I alpha 1 (Col1A1), fibronectin (Fn) and transforming growth factor-beta 1 (TGFβ1) in liver of MCD diet-fed mice (**Figure 6D**). Furthermore, the protein levels of Col1A1 and collagen type III alpha 1 (Col3A1) in the liver of MCD diet-fed mice were dramatically up-regulated, while theabrownin significantly decreased the levels of Col1A1 and Col3A1 (**Figures 6A–C**). Together, theabrownin reduced hepatic oxidative stress and fibrosis in MCD diet-fed mice.

### 3.4 Theabrownin increases the expression of FGF21 and reduces the phosphorylation of p38 in MCD diet-fed mice

**Figure 7:**
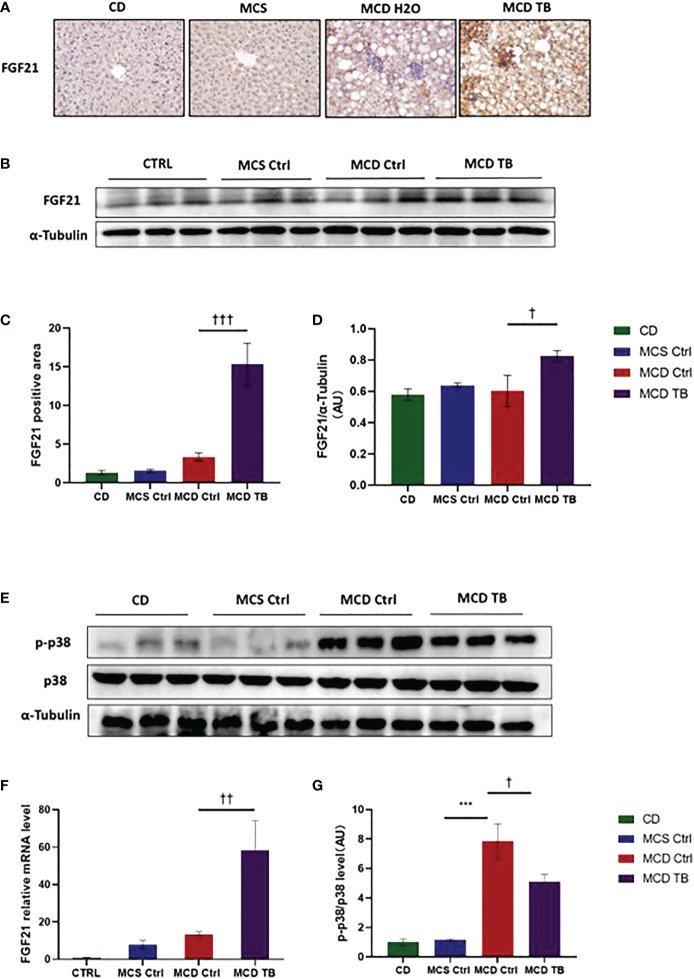
Theabrownin increases the expression of FGF21 and reduces the phosphorylation of MAPK p38 in MCD diet-fed mice. **(A)** IHC for FGF21. **(B)** FGF 21 protein levels from western blot. **(C)** Quantification of IHC positive area for FGF21. **(D)** Quantification of FGF 21 protein levels from western blot. **(E)** p38 protein level and p38 phosphorylation. **(F)** FGF21 mRNA level. **(G)** Quantification of p38 phosphorylation (normalized by total p38). AU, arbitrary units. n=5-11 mice per group. The data are mean ± s.e. (error bars). ***p < 0.001, MCS Ctrl vs. MCD Ctrl. †, p<0.05, ††, p<0.01, †††, p<0.001 MCD Ctrl vs. MCD TB.

p38, which is a mitogen-activated protein kinase (MAPK), is responsive to stress stimuli. p38 is a downstream molecule of FGF21 that can inhibit p38 phosphorylation (34). In order to uncover the therapeutic mechanism of theabrownin in treating NASH, we measured the levels of FGF21 mRNA and protein, as well as the phosphorylation of p38 in the liver. We found that the levels of FGF21 mRNA and protein in the liver of MCD diet-fed mice might be a little higher than that of MCS diet-fed mice (**Figures 7 A, F**), but there was no statistical difference. Theabrownin significantly increased the expression of FGF21 in the liver of MCD diet-fed mice, which can be seen by the results of FGF21 mRNA level, protein level, and immunohistochemical staining (**Figures 7A–F**). Meanwhile, theabrownin can reduce the high phosphorylation level of p38 induced by MCD diet in liver (**Figures 7E,G**)

## 4 Discussion

NASH is characterized by steatosis, inflammation, and fibrosis of the liver (6, 35). The progress of NASH is accompanied by the deposition of lipids in the liver, which leads to lipotoxicity (36, 37). In this study, we used MCD diet to induce NASH mouse model. As an experimentally common NASH model, the disease phenotypes of MCD-induced NASH model are different when compared with patients with NASH. However, MCD-induced NASH model is very similar to human NASH histology (38). Like previous studies, we found that mice fed with MCD showed weight and liver weight loss (39, 40), but theabrownin could not reverse these changes. Although MCD diet can mimic the pathological changes of human NASH well, it is different from obesity-induced NASH. MCD-fed mice show lower serum triglyceride (39, 41), which was confirmed in this study. Previous studies have shown that theabrownin can improve lipid metabolism and therefore reduce the high triglyceride concentration in liver and serum of mice induced by high fat or high-sugar diet (18, 42, 43). Interestingly, MCD-fed mice showed high liver lipid deposition but low expression of ACC, FASN and SREBP1c in liver. It indicated that rather than the increase of *de novo* lipogenesis in liver, fats from adipose tissue lipolysis overflow to liver may contribute to the high TG levels in liver of MCD diet-fed mice (22). Consistently, in our study, almost no white adipose tissue was found in mice fed with MCD diet. It suggested that adipose tissue lipolysis may happen in MCD diet fed mice. Moreover, theabrownin had no effect on liver lipid deposition and the expressions of lipogenic genes.

Inflammation plays a vital role in the occurrence and development of NAFLD/NASH (6, 44). Liver inflammation can activate and recruit inflammatory cells like macrophages to infiltrate liver, which in turn aggravates inflammatory responses (45). Therefore, reducing liver inflammation plays a key role in preventing the progression of NAFLD/NASH (44, 46). Other studies show that tea extract can improve the inflammation of NAFLD induced by high-fat diet (47). Moreover, Both Fuzhuan brick tea and theabrownin from Fuzhuan brick tea showed beneficial effects on obesity (23, 48–50). Although theabrownin has been reported to have beneficial effects on the improvement of obesity-related inflammatory diseases, most animal models used are limited to steatosis without obvious inflammation and NASH progression. In the current study, theabrownin has been shown to ameliorate liver injury and reduce inflammatory response, including reducing macrophage and neutrophil infiltration, inhibiting activation of NF κ B and JNK signaling pathways, and decreasing expression of inflammatory cytokines in MCD diet-fed mice. Oxidative stress is closely related to immune cell response. NASH is characterized by adaptive immune cell infiltration in the liver and the presence of circulating antibodies against antigens derived from oxidative stress (51). In NAFLD/NASH, lipid peroxidation which produces oxidized phospholipids mainly contributes to oxidative stress in liver. MDA is one of the final products of polyunsaturated fatty acid peroxidation. The increase of free radicals will lead to excessive production of MDA. MDA levels are often referred to as markers of oxidative stress. High levels of MDA were associated with hepatic steatosis and inflammation in NAFLD/NASH (52). In our study, MCD diet significantly enhanced the levels of ROS and MDA in mice, while theabrownin apparently reversed the elevation of ROS and MDA. Hepatic chronic oxidative stress and inflammation are important factors to advance the transition from NAFLD to NASH *via* activating hepatic stellate cells and promotes fibrosis (53, 54). TGFβ-1 is the most potent fibrogenic cytokine and a key driver of HSC activation and liver fibrosis. In NASH, TGFβ-1 stimulates the expression of α-SMA, which increase the levels of extracellular matrix proteins such as Fn and collagens, accelerating hepatic fibrosis (55). The ERK signaling pathway plays a key role in regulating the main phenotypic response of fibroblasts, driving liver fibrosis by targeting HSC (56). In liver macrophage, ERK stimulation the secretion of TGFβ-1 to activate HSC (57). We found MCD diet increased phosphorylation of ERK and levels of α-SMA, Col1A1, and Col3A1, promoting fibrosis in liver. Theabrownin remarkably reduced phosphorylation level of ERK and the levels of TGFβ, α-SMA, Col1A1, and Col3A1, as well as fibrosis induced by MCD diet.

FGF21 is an important regulator of energy homeostasis and a potential therapeutic target for metabolic diseases. FGF21 plays many beneficial metabolic roles such as increasing energy expenditure, β-oxidation, adiponectin secretion, and improving insulin resistance (58–61). FGF21 is an important potential target for the treatment of NAFLD/NASH. Previous studies have shown that FGF21 knockout or inhibitor of FGF21 can exacerbate the development of NAFLD/NASH (10, 62, 63), while pharmacological administration of FGF21 can effectively improve NAFLD/NASH (14, 64, 65). However, under pathological conditions of NAFLD/NASH, the expression of FGF21 would be increased in liver, which may be due to the "FGF21 resistance" phenomenon caused by the body's desire to improve the disease state by increasing FGF21. Just like in some patients with type 2 diabetes, the serum insulin level increases. We found in MCD diet-fed mice, the level of FGF21 was increased but not significantly compared to MCS diet-fed control mice. Theabrownin increased the level of FGF21 in MCD diet-fed mice. p38, which is a downstream molecule of FGF21, is activated in the livers of mouse models of obesity. It has been demonstrated that p38 might have a regulatory role in hepatic gluconeogenesis and lipogenesis (66, 67). Here, we found MCD diet stimulated p38 phosphorylation, which indicated that p38 activation was increased in NASH mouse model. A study showed that pharmacological administration of sulforaphane can increase the expression of FGF21 and reduce the phosphorylation level of p38 in liver, thus improving nonalcoholic fatty liver disease (34). Pharmacological administration of FGF21 to vascular smooth muscle cells can prevent calcification of vascular smooth muscle cells by inhibiting p38 signaling pathway (68). Administering analogues of FGF21 to adipocytes reduced loss of mature adipocytes and decreased phosphorylation levels of p38 (69). In addition, administration of analogues of FGF21 can alleviate liver fibrosis and reduce the phosphorylation level of p38 in liver of NASH mice (70). In our study, we observed theabrownin decreased p38 activation in MCD diet-fed mice.

## 5 Conclusion

In this study, we found that theabrownin had no effect on MCD-induced liver steatosis, but it showed a beneficial effect on liver injury, inflammatory response, oxidative stress, and fibrosis. Further study showed that theabrownin increased the level of FGF21 and reduced the phosphorylation of p38 in liver of MCD diet fed mice.

## Data availability statement

The raw data supporting the conclusions of this article will be made available by the authors, without undue reservation.

## Ethics statement

The animal study was reviewed and approved by Subcommittee of Experimental Animal Ethics, Shandong University (Permission number: SYKX20200022).

## Author contributions

QZ and QL conducted most of experiments. YZ, ZL, and CB participated some experiments. XG wrote most of this article. HW wrote some sections. XG and XZ made the final editing. All authors contributed to the article and approved the submitted version.
